# Biportal Endoscopic Foraminotomy with Unilateral Screw Fixation Using a Dynamic Rod for Radiculopathy Due to Osteoporotic Compression Fracture

**DOI:** 10.3390/jcm15134938

**Published:** 2026-06-25

**Authors:** Sang Youp Han, Sang Hyub Lee, Jae Won Jang, Yong Eun Cho, Choon Keun Park, Sang Won Lee

**Affiliations:** Department of Neurosurgery, Spine Center, The Leon Wiltse Memorial Hospital, Suwon 16480, Republic of Korea

**Keywords:** aged, fractures, compression, endoscopy, spinal fusion, lumbar vertebrae/surgery

## Abstract

**Objective**: Perform endoscopic surgery for radiculopathy caused by compression fractures and evaluate the results. **Methods**: A total of 20 patients who underwent biportal endoscopic foraminotomy and unilateral screw fixation using a dynamic rod for radiculopathy secondary to osteoporotic compression fractures were included in this study. All surgeries were performed between July 2021 and January 2025. Patient demographic data, operated level, length of hospital stay, intraoperative blood loss, and operative time were reviewed. Radiological follow-up included assessment of segmental kyphosis, scoliosis, subsidence, and adjacent-level fractures. Complications and pain patterns—separately evaluated for back pain and radiculopathy—were assessed using the visual analog scale (VAS) preoperatively and during follow-up. Only single-level cases were included. Patients with infections, significant stenosis, instability, tumors, prior revision surgery, multilevel pathology, or ambiguous symptoms were excluded. **Results**: The mean age of the patients was 78.8 years (range, 69–89 years), reflecting an elderly cohort. The mean follow-up period was 13.0 ± 11.9 months (range, 1–41 months). The mean operative time was 164.8 ± 25.7 min, and the mean hospital stay was 10.2 ± 4.6 days (range, 4–25 days). The mean intraoperative blood loss was 126.5 ± 77.6 mL (range, 50–400 mL). One female patient developed postoperative pneumonia, which resolved after appropriate treatment; no other medical complications were observed. Radiculopathy improved significantly immediately after surgery and continued to improve during follow-up. Back pain also improved, but tended to persist to a mild degree. Radiologic evaluation revealed no significant changes in segmental lordosis, and there were no cases of subsidence, scoliosis, or symptomatic screw loosening during the available follow-up period. **Conclusions**: Biportal endoscopic foraminotomy with unilateral screw fixation may be an effective solution for radiculopathy caused by compression fractures.

## 1. Introduction

Compression fractures are common lesions in the elderly. When accompanied by an inferior endplate fracture, bone fragments can form at the beak point, causing severe radiculopathy through direct compression of the nerve root [1].

However, elderly patients with such fractures often have poor general health and multiple comorbidities, which increase the risk of complications when using conventional surgical methods such as microscopic laminectomy or lumbar fusion [2]. Furthermore, decompression through endoscopic foraminotomy alone may result in residual pain and postoperative instability due to facet resection.

In this context, combining endoscopic foraminotomy with a dynamic rod may serve as an effective alternative. The present study aimed to evaluate the incidence of complications and the postoperative pain trajectory in elderly patients who underwent this combined procedure.

## 2. Materials and Methods

From July 2021 to January 2025, patients who underwent single level biportal endoscopic foraminotomy with dynamic rod fixation at our hospital were retrospectively reviewed. A total of 20 patients presented with sudden-onset back pain and radiating leg pain secondary to recent osteoporotic compression fractures. All patients had failed conservative management, including medication, physiotherapy, selective nerve root blocks, and bed rest. Patients with infections, significant stenosis, instability, tumors, revision surgery, multilevel pathology, or ambiguous symptoms were excluded. The presumed mechanism of radiculopathy was posterior displacement of a bony fragment from the fractured inferior endplate into the foramen, resulting in ventral compression of the exiting nerve root. During standing or ambulation, further collapse of the fractured vertebral body may aggravate posterior fragment migration and increase irritation of the nerve root, leading to severe radiating pain and walking difficulty. This mechanism was confirmed in all included patients by preoperative MRI and CT, and the radiologic compression level was required to correspond with the patient’s dermatomal symptoms. Patients with ambiguous symptoms or other potential causes of radiculopathy were excluded.

Demographic characteristics, operated level, length of hospital stay, intraoperative blood loss, and operative time were recorded. Pain was assessed using the visual analog scale (VAS) preoperatively, immediately postoperatively, and at 1, 3, and 6 months, as well as at ≥1 year at the last follow-up. Pain was evaluated separately for back pain (including operative site pain) and radiating leg pain. Radiological follow-up included assessment of segmental lordosis, subsidence, scoliosis, screw loosening, and adjacent-level fractures. Segmental lordosis was measured as the Cobb angle at the operated level, calculated from standing lateral radiographs. Subsidence was defined as a decrease in disc height of ≥2 mm compared with the immediate postoperative measurement. Screw loosening was defined as the presence of a radiolucent zone >1 mm around the screw on follow-up radiographs or CT scans.

Complications were categorized as medical (e.g., pneumonia, acute kidney injury) or surgery-related (including incidental durotomy, postoperative hematoma, paresthesia, and weakness) and were identified using postoperative dynamic radiographs, computed tomography (CT), magnetic resonance imaging (MRI), and medical records.

Continuous variables are presented as mean ± standard deviation. Changes in VAS scores between the preoperative and postoperative time points were analyzed using the Wilcoxon signed-rank test because of the small sample size and ordinal nature of the VAS score. For each comparison, only patients with available paired data were included. A *p*-value < 0.05 was considered statistically significant.

This study was approved by the Institutional Review Board of Leon Wiltse Memorial Hospital (IRB No. 2025-W01). The requirement for informed consent was waived by the Institutional Review Board because of the retrospective design of the study. Data was analyzed using SPSS version 28.0 (SPSS Inc., Chicago, IL, USA).

### 2.1. Surgical Methods

The procedure was performed under general or epidural anesthesia with the patient in the prone position on a radiolucent table. The operative level was confirmed using fluoroscopy. Two ipsilateral portals were created under fluoroscopic guidance. The cranial portal was used as the viewing portal, and the caudal portal was used as the working portal. The portals were placed approximately 1 cm lateral to the lateral border of the pedicle, along the isthmus line above and below the target disc space.

After serial dilatation and soft tissue dissection, the endoscope was introduced through the viewing portal. The anatomical landmarks, including the isthmus, superior articular process, transverse process, and facet joint, were identified. Lateral foraminotomy was then performed using a high-speed drill and Kerrison punch. The tip of the superior articular process and part of the facet joint were removed to widen the foramen and expose the ligamentum flavum. The ligamentum flavum was detached and removed to identify the exiting nerve root.

**Figure 1:**
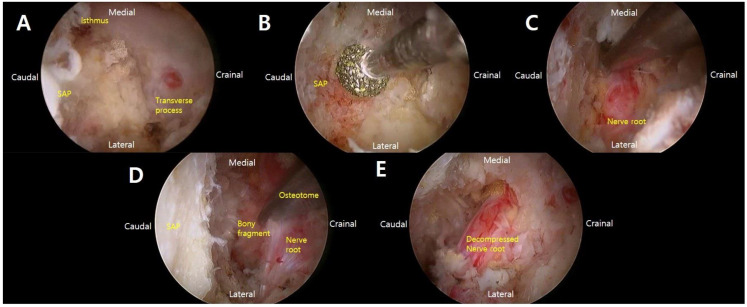
Endoscopic view of biportal endoscopic foraminotomy and monosegment screw fixation. (**A**) Two portals are created for endoscope insertion and identification of anatomical landmarks. (**B**) Drilling is performed at the tip of the superior articular process and extended toward the isthmus. (**C**) The ligamentum flavum is removed using a Kerrison punch or curette. (**D**) After identifying the exiting nerve root, bony fragments in the ventral area are removed to achieve decompression. (**E**) Adequate decompression is confirmed, a Hemovac drain is placed, and the procedure is completed.

After the exiting nerve root was confirmed, decompression was continued along the course of the nerve root. The retropulsed bony fragment arising from the fractured inferior endplate was identified at the ventral aspect of the exiting nerve root and removed using a curette, pituitary forceps, or Kerrison punch. Adequate decompression was confirmed when the exiting nerve root was freely mobilized and no residual ventral compression was observed endoscopically (Figure 1).

After completion of the endoscopic decompression, the skin incision was slightly extended to allow percutaneous pedicle screw insertion. Pedicle screws were inserted unilaterally at the affected segment under fluoroscopic guidance. Cement augmentation was performed through the fenestrated pedicle screws in all patients to improve screw purchase in osteoporotic bone. Care was taken to avoid cement leakage, especially through the fracture line.

**Figure 2:**
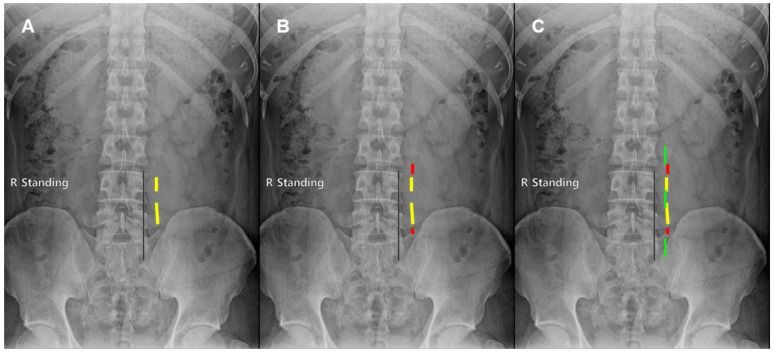
Stepwise procedure of biportal endoscopic foraminotomy and monosegment screw fixation on radiographic view. (**A**) Biportal endoscopic foraminotomy is performed with two small incisions (yellow lines). The black line indicates the pedicle lateral margin. (**B**) After nerve root decompression through foraminotomy, the incision is slightly extended to allow for percutaneous pedicle screw fixation (red lines). (**C**) After pedicle screw insertion, the incision is further extended and the fascia is incised to insert the dynamic rod (green lines).

After screw placement and cement augmentation, a dynamic rod was inserted and connected to the unilateral pedicle screws. The rod was secured to provide segmental support while allowing limited motion. After final fluoroscopic confirmation of screw and rod position, the endoscope was reintroduced to inspect the decompression site and remove any residual hematoma. A Hemovac drain was placed, and the fascia, subcutaneous tissue, and skin were closed in layers (Figure 2).

### 2.2. Illustrated Case

**Figure 3:**
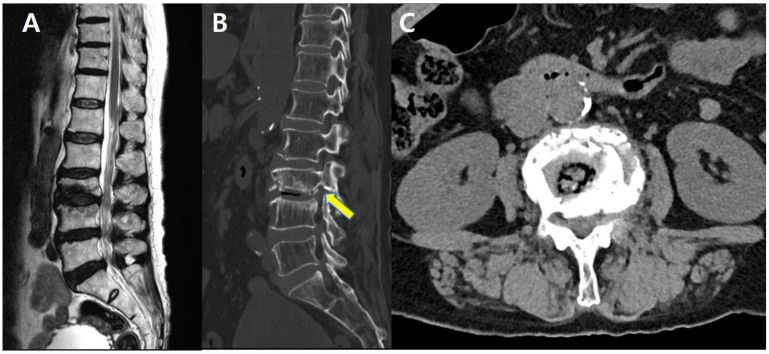
An 82-year-old female patient complains of severe radiating pain in the left leg due to an L3 osteoporotic compression fracture. (**A**) MRI shows a fracture of the L3 inferior end plate. (**B**) At the beak point, the fractured bone is obstructing the foramen (yellow arrow). (**C**) CT axial images also reveal the fracture.

An 82-year-old woman presented with sudden-onset low back pain and radiating pain in the left leg. Magnetic resonance imaging (MRI) and computed tomography (CT) demonstrated an inferior endplate fracture of L3 with a retro pulsed fragment irritating the L3 nerve root (Figure 3). Conservative management, including a selective L3 nerve root block, failed, and she was unable to ambulate because of severe pain.

**Figure 4:**
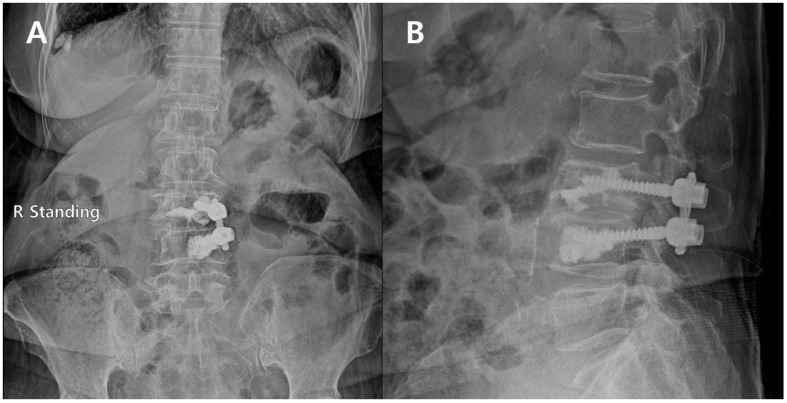
Postoperative L-spine X-ray shows cement-augmented mono screw fixation. (**A**) AP view on X-ray. (**B**) Lateral view on X-ray.

Comorbidities included hypertension, diabetes mellitus, and chronic kidney disease. Given her age and medical status, we planned biportal endoscopic foraminotomy combined with mono-segment pedicle screw fixation with cement augmentation (Figure 4). Operative time was approximately 2 h, and the estimated blood loss was 50 mL.

Postoperatively, the patient’s radicular symptoms improved immediately, and both back pain and wound pain were mild. She was discharged on postoperative day 4. At scheduled follow-up visits, she ambulated independently without recurrent severe pain.

ChatGPT (OpenAI) was used only for language editing and improvement of manuscript readability. No AI tools were used for data analysis, interpretation of results, or figure generation. The authors reviewed and edited all AI-generated content and take full responsibility for the content of this publication.

## 3. Results

### 3.1. Demographics

From July 2021 to January 2025, patients who underwent surgery in our hospital were investigated for single-level compression fracture with radiculopathy. A total of 20 patients were enrolled and underwent endoscopic foraminotomy and mono-segment fixation. The mean age of the patients was 78.8 ± 4.9 years (69–89 years). The patients consisted of 4 males and 16 females, with a higher proportion of females. The most common surgical level was L4–5 (9 cases, 45%). The mean follow-up period was 13.0 ± 11.9 months. The operation time was 164.8 ± 25.7 min. The hospital stay was 10.2 ± 4.6 days (4–25 days). The blood loss was 126.5 ± 77.6 mL (50–400 mL) (Table 1). A patient-level summary is provided in Table 2. The table includes age, sex, operative level, affected nerve root, side, radiologic compression mechanism, follow-up duration, and complications. Cement augmentation was performed in all patients.

### 3.2. Pain

**Figure 5:**
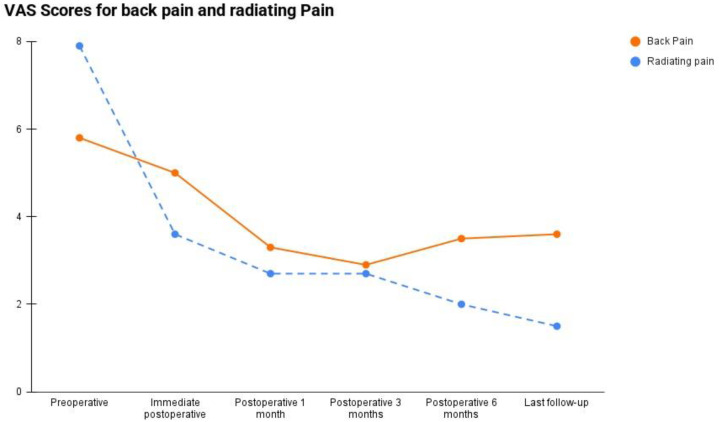
Visual analog scale (VAS) scores for back pain and radiating pain over time.

The mean preoperative VAS scores were 7.9 ± 1.4 for radiating pain and 5.8 ± 1.1 for back pain. Immediately after surgery, the scores improved to 3.6 ± 0.9 and 5.0 ± 0.9, respectively. At 1 month postoperatively, the scores further decreased to 2.7 ± 1.1 for radiating pain and 3.3 ± 0.8 for back pain. At 3 months, the scores were 2.7 ± 1.6 and 2.9 ± 0.9, and at 6 months, 2.0 ± 1.1 and 3.5 ± 0.5, respectively. At the final follow-up, the mean VAS scores were 1.5 ± 1.1 for radiating pain and 3.6 ± 1.4 for back pain (Table 3, Figure 5).

Compared with the preoperative values, radiating pain improved significantly at all postoperative time points: immediately postoperatively, 1 month, 3 months, 6 months, and the last follow-up (all *p* < 0.001). Back pain also improved significantly compared with the preoperative value, although the immediate postoperative improvement was smaller (immediate postoperative, *p* = 0.035; 1 month, 3 months, and 6 months, all *p* < 0.001; last follow-up, *p* = 0.002).

### 3.3. Radiologic Outcomes

The mean segmental lordosis decreased by 0.13°, with a standard deviation of 5.0°. In most patients, there was no significant change between pre- and postoperative measurements; however, in one patient (82-year-old female), a decrease of approximately 10° was noted at 42 months postoperatively. No cases of subsidence, scoliosis were identified according to our definition, and screw loosening was observed in only one case, which was asymptomatic and involved a single screw (Table 4).

Because the follow-up duration was variable, we performed an additional subgroup analysis of patients with at least 12 months of follow-up. Nine patients met this criterion, with a mean follow-up duration of 23.2 ± 10.4 months (range, 12–41 months). In this subgroup, no symptomatic screw loosening, subsidence, scoliosis, or revision surgery was observed. Adjacent-level compression fractures occurred in two patients and were treated with vertebroplasty.

### 3.4. Complications

Pneumonia occurred in one patient (89-year-old female, general anesthesia, history of hypertension, diabetes mellitus, atrial fibrillation, and chronic kidney disease) after surgery; the patient fully recovered after several days of treatment. No other significant medical complications were observed in the remaining patients. In addition, adjacent-level compression fractures occurred in two cases, at 31 and 34 months postoperatively, respectively, both of which were treated with vertebroplasty. One case of radiological screw loosening was noted without associated symptoms (Table 5).

Regarding functional recovery and reoperation-related outcomes, all patients were able to ambulate after surgery during follow-up. No patient required revision surgery, additional decompression, or additional fixation at the index level. Two patients underwent vertebroplasty for subsequent adjacent-level compression fractures, which were regarded as subsequent procedures rather than revision surgery for the index operation.

Cement augmentation was performed in all patients to improve screw purchase in osteoporotic bone. Postoperative osteoporosis medication was also prescribed in all patients, including romosozumab, teriparatide, or denosumab, according to the patient’s clinical condition and physician preference.

## 4. Discussion

Osteoporosis-related compression fractures are common in elderly patients, often causing severe pain and mobility limitations, which may lead to secondary complications. For general compression fractures, vertebroplasty can provide effective pain relief and functional improvement [3,4,5]. However, when fractures involve the inferior endplate, patients may present with both back pain and radiculopathy. Approximately 41% of compression fracture patients have inferior endplate involvement; in such cases, bone fragments can compress the exiting nerve root at the foramen, producing severe radiculopathy [6,7]. This pain may be so intense that it restricts ambulation, increasing the risk of pneumonia, ileus, constipation, and other complications.

In our treatment algorithm, vertebroplasty or kyphoplasty was considered more appropriate when axial back pain from the compression fracture was the dominant symptom without definite foraminal nerve root compression. Endoscopic decompression alone may be considered when foraminal compression is limited and sufficient decompression can be achieved without substantial facet resection. However, in patients with severe radiculopathy and walking difficulty caused by direct foraminal compression from an inferior endplate fragment, vertebral augmentation alone may not address the primary pain generator. When adequate decompression requires removal of the bony fragment and partial facetectomy, unilateral fixation with a dynamic rod may be useful to maintain foraminal height, reduce postoperative micromotion, and prevent iatrogenic instability while avoiding the invasiveness of conventional fusion.

Although patients with overt preoperative instability were excluded, fixation was considered necessary because adequate lateral foraminotomy required partial facetectomy and removal of the compressive bony fragment. In osteoporotic patients with recent compression fractures, decompression alone may increase segmental micromotion, promote further foraminal collapse, or result in iatrogenic instability. Therefore, unilateral screw fixation with a dynamic rod was used to provide temporary segmental support, maintain foraminal height, and reduce micromotion until fracture healing and bony remodeling occurred.

Foramen lesions can be managed conservatively with selective nerve root block (SNRB), but in many cases the root irritation is severe and resistant to injections or medication. Traditionally, surgical options such as TLIF, PLIF, or microscopic foraminotomy have been used [8,9,10,11,12]. However, these procedures often involve considerable blood loss, prolonged operative time, and significant postoperative pain, increasing the risk of severe medical complications in elderly patients. Moreover, fusion surgery for inferior endplate fractures frequently results in non-union and unsatisfactory outcomes [13,14].

With the advancement of endoscopic surgery, minimally invasive approaches have become available, reducing complications and improving safety in elderly patients [15,16]. In cases of radiculopathy from compression fractures, foraminotomy combined with unilateral screw fixation using a dynamic rod can support the facet joint during healing and provide rapid symptom improvement without significant postoperative pain or complications. This technique has also shown consistent benefits during long-term follow-up.

In the present study, the cohort was predominantly elderly (mean age, 78.8 years) and female, with the most common operative level being L4–5 (45% of cases). Compared with conventional fusion procedures, endoscopic surgery achieved favorable outcomes in terms of intraoperative blood loss, operative time, and hospital stay. The mean blood loss and operative time were slightly increased by a few outlier cases; however, in most patients, the operative time was approximately 2 h and blood loss ranged from 50 to 100 mL. The mean hospital stay was 4–7 days, except for one patient requiring 25 days of hospitalization for pneumonia treatment. Overall, medical complications were rare. The only pneumonia case occurred in an 89-year-old woman with an ASA (American Society of Anesthesiologists physical status classification system) score of 3 and multiple comorbidities, including hypertension, diabetes mellitus, atrial fibrillation, and chronic kidney disease.

Postoperative pain outcomes were favorable. Severe preoperative radiculopathy improved rapidly after surgery, and operative site pain was milder than that typically observed after conventional microscopic fusion. This pattern persisted throughout follow-up: some degree of back pain remained over time, but radiculopathy was minimal at the last follow-up. No revision surgeries were required. This difference between radiating pain and back pain may be explained by the different pain generators. Radiating pain was mainly caused by direct foraminal compression of the exiting nerve root by the inferior endplate fragment; therefore, endoscopic decompression and segmental support could produce rapid symptom relief. In contrast, back pain was likely multifactorial, including fracture-related vertebral body pain, residual axial pain from the osteoporotic compression fracture, paraspinal muscle injury, and postoperative wound pain. Therefore, mild residual back pain may persist even after adequate neural decompression and fixation. These findings suggest that this procedure may improve radiculopathy more reliably than fracture-related axial back pain.

Radiological outcomes also demonstrated stability over a mean follow-up of 13 months (range, 1–41 months). Spinal alignment was preserved without significant deformity, and segmental lordosis showed no meaningful change. No subsidence was observed, possibly due to the use of dynamic rods and the limitation to single-level surgery. Concerns about scoliosis from unilateral screw fixation were not substantiated, as no cases occurred. Most patients underwent cement augmentation, which likely contributed to the absence of screw loosening or subsidence; however, it may have been a factor in the two cases of adjacent-level compression fractures observed.

No surgery-related complications such as dural tear, neural damage, cement leakage, excessive bleeding, or infection were encountered.

Several technical points may enhance outcomes with this approach. First, the rationale for using a dynamic rod was to avoid an overly rigid construct in elderly osteoporotic patients. Rigid fixation may increase stress concentration at the bone–screw interface and adjacent segments, potentially contributing to screw loosening or subsequent compression fractures. In the present procedure, the anterior column and disc space were largely preserved, whereas partial unilateral facetectomy during lateral foraminotomy was the main surgically induced destabilizing factor. Therefore, a dynamic rod was selected to provide temporary posterior support, maintain foraminal height, and reduce micromotion while allowing limited segmental motion and load sharing. Cement augmentation was used in all patients to improve screw purchase in osteoporotic bone, and careful injection was required to avoid cement leakage through the fracture line. However, because this study did not directly compare dynamic and rigid rods, the potential benefit of the dynamic rod should be interpreted as a biomechanical rationale rather than definitive clinical superiority. Second, during foraminotomy, decompression should be sufficient to remove the ventral bony fragment and release the exiting nerve root. Because unilateral fixation is performed after decompression, adequate removal of the compressive fragment and partial facetectomy can be performed without excessive concern for immediate postoperative instability. In our experience, following the exiting nerve root laterally toward the transverse process membrane is useful for confirming adequate foraminal decompression.

This study has several limitations. The sample size was small (n = 20), there was no comparison group undergoing fusion or microscopic foraminotomy, and no direct comparison between dynamic and rigid rods was performed. Because this was a retrospective single-arm case series, the present study cannot determine whether this combined approach is superior to conventional fusion, vertebral augmentation, or endoscopic decompression alone. Therefore, the results should be interpreted as preliminary findings demonstrating the feasibility and potential clinical usefulness of this technique in carefully selected patients, rather than evidence of superiority over other treatment options. Further large-scale, comparative studies are warranted to validate these findings. However, the follow-up duration was variable, ranging from 1 to 41 months. Therefore, conclusions regarding long-term stability, screw loosening, subsidence, and adjacent-level fractures should be interpreted cautiously. Although no symptomatic screw loosening, subsidence, scoliosis, or revision surgery was observed in patients with at least 12 months of follow-up, further studies with longer and more uniform follow-up are required.

ODI, formal patient satisfaction scores, and quantitative analgesic consumption were not routinely collected because of the retrospective design of this study. Therefore, these functional outcomes could not be analyzed and should be evaluated in future prospective studies.

Although osteoporosis medication was prescribed in all patients, detailed patient-level data regarding the specific medication, treatment duration, and compliance were not consistently available because of the retrospective design. Therefore, the effect of osteoporosis treatment on screw stability and adjacent-level fracture risk could not be analyzed.

## 5. Conclusions

Biportal endoscopic foraminotomy with unilateral screw fixation may be a feasible treatment option for managing radiculopathy caused by osteoporotic compression fractures in carefully selected elderly patients.

## Figures and Tables

**Table 1:** Clinical data of compression fracture with radiculopathy.

	Compression Fracture with Radiculopathy
No. of patients	20
Sex	
Male	4
Female	16
Age (years)	78.8 ± 4.9 (69–89)
Most common level	L4/5 (9 cases, 45.0%)
Average follow-up period (months)	13.0 ± 11.9 (1–41)
Operation time (min)	164.8 ± 25.7
Hospital day (days)	10.2 ± 4.6 (4–25)
Blood loss (mL)	126.5 ± 77.6 (50–400)

**Table 2:** Patient-level summary of osteoporotic compression fracture with radiculopathy.

Case	Age/Sex	Operative Level	Side	Affected Nerve Root	Compression Mechanism	Follow-Up	Complication
1	69/F	L4–5	Lt	L4 exiting root	Foraminal compression by inferior endplate bony fragment	11 mo	None
2	74/F	L4–5	Rt	L4 exiting root	Foraminal compression by inferior endplate bony fragment	3 mo	None
3	82/F	L3–4	Lt	L3 exiting root	Ventral compression by retropulsed inferior endplate fragment	41 mo	Adjacent fracture at 31 mo
4	72/F	L4–5	Lt	L4 exiting root	Foraminal compression by inferior endplate bony fragment	12 mo	None
5	78/F	L2–3	Rt	L2 exiting root	Foraminal compression by inferior endplate bony fragment	9 mo	None
6	79/F	L1–2	Rt	L1 exiting root	Foraminal compression by inferior endplate bony fragment	13 mo	None
7	78/M	L1–2	Lt	L1 exiting root	Foraminal compression by inferior endplate bony fragment	30 mo	None
8	76/F	L4–5	Lt	L4 exiting root	Foraminal compression by inferior endplate bony fragment	7 mo	None
9	80/F	L3–4	Rt	L3 exiting root	Ventral compression by retropulsed inferior endplate fragment	36 mo	Adjacent fracture at 34 mo
10	81/F	L4–5	Lt	L4 exiting root	Foraminal compression by inferior endplate bony fragment	3 mo	None
11	80/F	L5–S1	Lt	L5 exiting root	Foraminal compression by inferior endplate bony fragment	3 mo	None
12	78/M	L4–5	Rt	L4 exiting root	Foraminal compression by inferior endplate bony fragment	20 mo	None
13	74/F	L3–4	Rt	L3 exiting root	Ventral compression by retropulsed inferior endplate fragment	15 mo	None
14	73/F	L3–4	Rt	L3 exiting root	Ventral compression by retropulsed inferior endplate fragment	24 mo	None
15	82/F	L3–4	Rt	L3 exiting root	Ventral compression by retropulsed inferior endplate fragment	1 mo	None
16	83/M	L4–5	Rt	L4 exiting root	Foraminal compression by inferior endplate bony fragment	3 mo	None
17	89/F	L4–5	Lt	L4 exiting root	Foraminal compression by inferior endplate bony fragment	18 mo	Pneumonia
18	84/M	L4–5	Rt	L4 exiting root	Foraminal compression by inferior endplate bony fragment	4 mo	None
19	79/F	L3–4	Lt	L3 exiting root	Ventral compression by retropulsed inferior endplate fragment	3 mo	Asymptomatic screw loosening
20	85/F	L2–3	Rt	L2 exiting root	Foraminal compression by inferior endplate bony fragment	3 mo	None

Lt, left; Rt, right. Cement augmentation was performed in all patients. The affected nerve root was determined based on the operative level, preoperative MRI/CT findings, and corresponding dermatomal symptoms. Adjacent fracture indicates a newly developed compression fracture at an adjacent or other vertebral level during follow-up.

**Table 3:** Visual analog scale (VAS) scores for back pain (including operative site pain) and radiating pain in patients with compression fracture and radiculopathy.

Time Point	Radiating Pain	*p*-Value	Back Pain (Including Operative Site Pain)	*p*-Value
Preoperative	7.9 ± 1.4	Reference	5.8 ± 1.1	Reference
Immediate postoperative	3.6 ± 0.9	<0.001	5.0 ± 0.9	*p* = 0.035
Postoperative 1 month	2.7 ± 1.1	<0.001	3.3 ± 0.8	<0.001
Postoperative 3 months	2.7 ± 1.6	<0.001	2.9 ± 0.9	<0.001
Postoperative 6 months	2.0 ± 1.1	<0.001	3.5 ± 0.5	<0.001
Last follow-up	1.5 ± 1.1	<0.001	3.6 ± 1.4	*p* = 0.002

Values are presented as mean ± standard deviation. *p*-values were calculated using the Wilcoxon signed-rank test compared with the preoperative value. Only patients with available paired data were included in each comparison.

**Table 4:** Radiologic outcomes after biportal endoscopic foraminotomy with unilateral screw fixation.

Radiologic Parameter	Findings (n = 20)
Change in segmental lordosis (°)	−0.13 ± 5.0
Significant decrease (>10°)	1 case (82F, at 42 months)
Subsidence	None
Scoliosis	None

**Table 5:** Postoperative complications.

Type of Complication	Cases (n = 20)	Details/Management
Medical: Pneumonia	1 (5%)	89F, ASA 3, multiple comorbidities; recovered with conservative treatment
Radiologic: Screw loosening	1 (5%)	Asymptomatic, single screw, observed
Radiologic: Adjacent-level fracture	2 (10%)	At 31 & 34 months; treated with vertebroplasty

## Data Availability

The data presented in this study are not publicly available due to privacy and ethical restrictions. Data may be available from the corresponding author upon reasonable request and with permission from the Institutional Review Board.
